# Retraction: Road traffic deaths and injuries are under-reported in Ethiopia: A capture-recapture method

**DOI:** 10.1371/journal.pone.0341365

**Published:** 2026-01-22

**Authors:** The PLOS One Editors

After this article [1] was published, concerns were raised about the results reported in Table 2 and Table 3 and whether the capture-recapture formula was correctly implemented.

Specifically, the estimated number of deaths and injuries reported cannot be reproduced when using the unmatched and matched police and hospital data on deaths and injuries presented in Table 2 and Table 3. This concern was evaluated in consultation with one of *PLOS One*’s statistical advisors and an independent member of its Editorial Board, who advised there is a discrepancy between the results reported for estimated numbers of deaths and injuries in Tables 2 and 3 and the expected formula outputs given the values reported for unmatched police data, unmatched hospital data, and number of matches. A possible labeling error of the unmatched data columns in Tables 2 and 3 was considered as a potential explanation; however, the consulting Editorial Board member advised that this would not be consistent with all of the data reported in Table 3. Further to this, the Editorial Board member noted that the methods used to calculate the standard error and confidence intervals reported in Table 2 and Table 3 have not been provided and as such cannot be reproduced.

The *PLOS One* Editors reached out to the authors to discuss the concerns raised; however, PLOS has been unable to obtain the underlying data required to clarify the nature and extent of these issues. As such, the reliability and validity of the reported results is in question.

In light of these issues, the *PLOS One* Editors retract this article [1].

TA did not agree with the retraction. YB, AW, AAssrat, and AAssefa either did not respond directly or could not be reached.

## References

[1] Abegaz T, Berhane Y, Worku A, Assrat A, Assefa A. RETRACTED: oad traffic deaths and injuries are under-reported in Ethiopia: a capture-recapture method. PLoS One. 2014;9(7):e103001. doi: 10.1371/journal.pone.0103001 25054440 PMC4108419

